# Research Progress on Genetic Factors of Poultry Egg Quality: A Review

**DOI:** 10.3390/ani15243652

**Published:** 2025-12-18

**Authors:** Liu Yang, Yang Yang, Yadi Jing, Meixia Zhang, Min Zhang, Shuer Zhang, Chao Qi, Weiqing Ma, Muhammad Zahoor Khan, Mingxia Zhu

**Affiliations:** 1 College of Agriculture and Biology, Liaocheng University, Liaocheng 252000, China; 18769301701@163.com (L.Y.); yy0210012025@163.com (Y.Y.); 2410190103@stu.lcu.edu.cn (Y.J.); 2022404148@stu.lcu.edu.cn (M.Z.); mawq1994@163.com (W.M.); zahoorkhan@lcu.edu.cn (M.Z.K.); 2 Provincial Animal Husbandry Station, 68 Huaicun Street, Huaiyin District, Jinan 250022, China; 18866617369@163.com (M.Z.); zhangshuer128@163.com (S.Z.); lssqc@163.com (C.Q.)

**Keywords:** poultry egg quality, genome-wide association study (GWAS), quantitative trait loci (QTL), multi-omics, eggshell mineralization, follicular development, epigenetic regulation, heritability, albumen and yolk traits, genomic selection

## Abstract

Egg quality is a critical economic trait in poultry production, influencing consumer preference and production efficiency. The genetic and epigenetic regulation of egg quality involves complex biological pathways across various traits such as shell quality, albumen composition, and yolk biochemistry. This review synthesizes recent advances in the genetic, molecular, and epigenetic mechanisms that determine poultry egg quality. Specifically, it focuses on external traits such as eggshell strength, color, and thickness, and internal traits including albumen height, yolk composition, and the Haugh unit. Through genome-wide association studies (GWAS), quantitative trait loci (QTL) mapping, whole-genome sequencing (WGS), and multi-omics approaches, key candidate genes such as *OC-116*, *CALB1*, *CA2* (shell formation), *OVAL*, *SPINK5*, *SERPINB14* (albumen quality), and *FGF9*, *PIAS1*, *NOX5* (lipid metabolism) have been identified. These genes play a pivotal role in shell biomineralization, albumen protein regulation, and yolk lipid transport. This review also explores the heritability of these traits, emphasizing the challenges posed by polygenic architecture and the influence of environmental factors. Furthermore, it addresses the dynamic spatiotemporal regulation of egg quality traits, including epigenetic layers such as DNA methylation, histone modifications, RNA methylation, and post-translational protein modifications. This paper highlights the application of these findings to breeding programs via genomic selection, marker-assisted breeding, and epigenetic engineering approaches. Future directions for precision breeding and the development of functional eggs with enhanced quality are also discussed.

## Simple Summary

This review synthesizes recent progress on the genetic and epigenetic determinants of poultry egg quality. It covers external traits (shell thickness, color, strength) and internal traits (albumen height, yolk composition, Haugh unit), outlining how QTL/GWAS, WGS, and multi-omics have mapped candidate genes and pathways for shell biomineralization, albumen protein regulation, and yolk lipid transport. It summarizes heritability patterns across traits and ages, highlights regulatory biology of follicular development and calcium transport, and details epigenetic layers (DNA methylation, histone marks, RNA methylation, post-translational protein modifications). This paper emphasizes translation to breeding via genomic selection and marker-assisted strategies, while noting polygenic architecture and environmental interactions as key challenges. It concludes with prospects for integrative, single-cell, and epigenetic engineering approaches to develop precision breeding programs and value-added “functional” eggs.

## 1. Introduction

Poultry eggs are a globally consumed food source valued for their exceptional nutritional composition, including high-quality proteins, essential amino acids, fatty acids, vitamins, and minerals that contribute to human health and nutrition [1,2,3]. According to recent estimates, global egg production has expanded dramatically, rising by approximately 450% from 1961 to 2017—reaching around 80 million metric tons, according to FAO data [4,5]. Egg quality traits can be broadly categorized into external characteristics, such as shell strength, color, and shape, and internal characteristics, including albumen height, yolk color, and Haugh unit [6,7,8]. These traits influence consumer preference, market price, processing suitability, and embryo viability, making them critical selection targets in modern poultry breeding programs [9,10,11].

Over the past few decades, significant advances in quantitative genetics and molecular biology have deepened our understanding of the genetic architecture governing egg quality traits [12,13]. Traditional breeding methods based on phenotypic selection and family-based approaches have successfully improved key egg traits such as shell thickness, egg weight, albumen quality, and yolk pigmentation [2,14,15]. However, the emergence of molecular genetics tools such as genome-wide association studies (GWAS), quantitative trait loci (QTL) mapping, and marker-assisted selection (MAS) has revolutionized poultry breeding by identifying candidate genes and chromosomal regions associated with key egg quality traits [16,17,18,19,20,21,22]. For instance, genes related to shell formation (*OC-116*, *CALB1*, *CA2*), albumen quality (*OVAL*, *SERPINB14*, *SPINK5*) have been identified in various chicken breeds [23,24,25,26,27].

The advent of multi-omics platforms—including genomics, transcriptomics, proteomics, metabolomics, and, more recently, epigenomics and single-cell RNA sequencing—has enabled a systems-level understanding of egg formation [28,29,30,31,32]. These approaches have elucidated how transcription factors, signaling pathways (e.g., TGF-β, BMP, and Wnt), and post-translational modifications interact to regulate shell biomineralization, albumen protein folding, and yolk lipid transport [33,34,35]. Moreover, gene-editing tools such as CRISPR/Cas9 now offer the possibility of precisely modifying alleles that control egg traits without compromising animal welfare or productivity [36,37,38].

Despite substantial progress, important gaps remain. Most studies analyze single traits or isolated regulatory layers, making it difficult to connect heritability patterns, genetic architecture, and molecular pathways into a unified understanding of egg quality. Existing reviews often treat quantitative genetics, physiology, and omics evidence separately, without linking genetic variants to tissue-specific mechanisms or epigenetic regulation across the liver, ovary, oviduct, and shell gland [39,40,41,42,43,44]. The practical implications for genomic selection, marker-assisted breeding, and emerging epigenetic approaches also remain unclear. Given the polygenic and environmentally sensitive nature of egg quality traits, a coherent framework integrating genetic markers, molecular networks, and epigenetic modulation is still needed [39,40,41,42,43,44].

A comprehensive understanding of the genetic and non-genetic factors influencing egg quality is essential for sustainable genetic improvement. This review synthesizes recent advances in the genetic and molecular mechanisms of egg quality, examines egg formation through a multi-omics perspective, and identifies key regulatory genes and networks. By integrating genetic, molecular, and epigenetic insights, it provides a framework for precision breeding strategies and the development of functional eggs with improved quality.

## 2. Methodology for Literature Search

This review was developed through a structured and reproducible literature search aimed at identifying genetic, molecular, epigenetic, and heritability-related determinants of poultry egg quality. A systematic search was performed in four major scientific databases—Web of Science, Scopus, PubMed, and Google Scholar. Searches combined controlled vocabulary and free-text terms using Boolean operators. Core terms included: poultry egg quality, eggshell strength, albumen quality, yolk composition, heritability, GWAS, QTL mapping, genetic markers, multi-omics, epigenetic regulation, transcriptomics, proteomics, “metabolomics, CRISPR, and species-specific terms such as chicken, duck, goose, quail, and poultry. Reference lists of key papers and prior reviews were manually screened to identify studies not captured through the primary search. Only studies available in English were included to ensure consistent interpretation.

**Figure 1:**
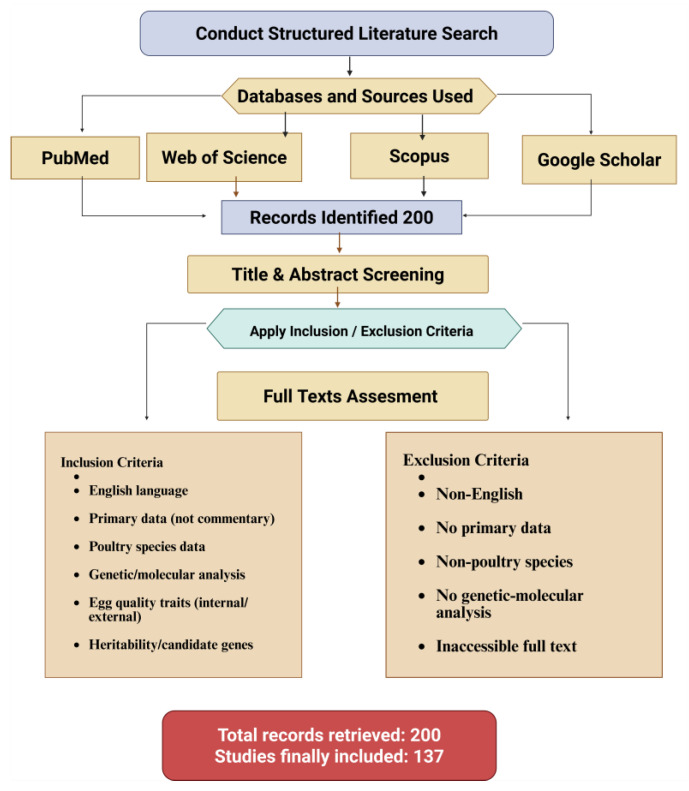
A flowchart of the literature search and article selection.

A total of 200 records were retrieved initially. These records were screened in two stages. First, titles and abstracts were reviewed to remove duplicates and exclude studies unrelated to egg quality or lacking genetic or molecular relevance. Full texts of the remaining papers were then assessed against predefined inclusion and exclusion criteria. Studies were included if they: (1) reported genetic, genomic, transcriptomic, proteomic, metabolomic, or epigenetic analyses of egg quality traits; (2) investigated external or internal egg-quality parameters; or (3) evaluated heritability or candidate genes in poultry species. Studies were excluded if they: (1) lacked primary data (e.g., commentaries, opinion pieces), (2) focused solely on non-poultry species without extractable poultry data, (3) provided no genetic or molecular analysis, or (4) were inaccessible in full text. After screening, 138 studies were retained for synthesis. The final body of evidence was organized thematically to cover shell quality, albumen traits, yolk composition, heritability, and epigenetic regulation. The literature screening process and inclusion/exclusion workflow are summarized in Figure 1.

## 3. Poultry Eggs Quality and Genetic Factors

The formation of poultry eggs is a complex, dynamic process influenced by multiple factors, among which genetic components play a pivotal role [45,46]. Genetic architecture research forms the foundation of understanding how shell, albumen, and yolk traits arise, and major advances have been achieved through molecular genetics tools. Early studies during the microsatellite marker era identified numerous quantitative trait loci (QTLs) using F_2_ hybrid populations through linkage analysis [13,47,48]. The subsequent development of SNP chip arrays and high-throughput sequencing technologies enabled genome-wide association studies (GWAS) to become a powerful tool for detecting genetic variations in livestock. For example, Zhang et al. [49], applied GWAS to identify SNPs and INDELs associated with duck egg quality traits. Similarly, the first GWAS in chickens using the Illumina 60K SNP chip revealed associations between specific genomic regions and egg quality traits [50]. Traits with high heritability—such as egg weight and shell strength—have shown significant improvements through genomic selection approaches [11].

Building on this architecture, recent work has shifted from simply identifying loci to interpreting how these loci contribute to specific physiological pathways. Recent studies have increasingly identified candidate genes and genomic loci associated with various egg quality traits, supported by genome-wide analyses and functional [51]. Zhang et al. [52] reported 36 candidate genes linked to lipid metabolism, protein synthesis, water content, and embryonic development. Functional annotation suggested that genes such as *FGF9*, *PIAS1*, *FEM1B*, *and NOX5* regulate lipid biosynthesis, whereas *AP3S2*, *HSPA4*, *CABP7*, *PPP2CA*, *and UBE2B* participate in protein folding and modification. Genes including *CSF2*, *GDF9*, *RAPGEF6*, and *PAQR5* influence reproductive development, while *MICU2*, *ITGA11*, *WDR76*, *ANPEP*, and *P4HA2* contribute to yolk texture and moisture retention [52]. This functional grouping highlights that egg-quality genes operate through distinct biological modules—minerals, proteins, lipids, and developmental signaling—rather than acting in isolation.

Additional GWAS work has deepened this functional understanding. Zhao et al. [53], identified 148 SNPs and 32 candidate genes influencing egg number, many associated with hormone regulation and follicle growth (e.g., *NELL2*, *KITLG*, *GHRHR*, *NCOA1*). A notable genomic region on GGA5 containing YY1 and WDR25 represents a strong molecular marker for laying performance [53]. Whole-genome sequencing (WGS) studies have further refined variant detection, and research on protein modifications highlights post-translational regulation—an emerging dimension in egg-quality biology [54,55,56,57]. Despite these advances, the genetic improvement of egg quality remains challenging due to the relatively low heritability of some traits and the confounding effects of intensive selection for egg production.

Heritability (h^2^) quantifies the proportion of phenotypic variance explained by genetics, and understanding h^2^ is essential for interpreting how genetic findings translate into breeding outcomes. However, heritability estimates can vary depending on several factors, including environmental conditions, population size, species differences, measurement methods, and the quality of phenotypic records [58]. The method used for heritability estimation also influences the results: while traditional multi-trait animal models can accurately decompose variance components, genomic approaches estimate heritability using SNP markers. These genomic estimates have shown high concordance with traditional models, particularly for traits such as egg weight [58].

Current literature indicates that traits such as egg weight, shape index, shell color, shell thickness, and Haugh units generally exhibit moderate to high heritability [11,49]. In contrast, certain traits, like yolk color, display low heritability due to their strong dependence on dietary components, particularly carotenoid levels in feed [59]. Importantly, heritability patterns reveal constraints: traits with high h^2^ may still show antagonistic genetic correlations with production traits such as egg number [60]. These relationships underscore the polygenic and environmentally influenced nature of egg-quality traits. Given the polygenic nature of egg quality and the limited understanding of its underlying molecular mechanisms, further studies are needed. Investigating the egg formation process, component biosynthesis, and associated metabolic pathways may yield new insights into the genetic regulation of egg quality traits.

### 3.1. Dynamic Process of Egg Formation in Poultry

The poultry egg consists of several components, including the stratum corneum, mineralized eggshell, shell membrane, egg white, yolk membrane, and yolk, from the outermost to the innermost layers. Although the architecture is well characterized, recent work emphasizes that each structural layer reflects the coordinated action of genetic pathways controlling mineralization, protein secretion, and lipid transport [61,62]. Egg formation occurs primarily within the fallopian tubes, but it also involves three key processes: (i) hepatic synthesis and transport of yolk precursors, (ii) follicular development and hierarchical selection in the ovary, and (iii) calcium ion homeostasis that drives eggshell calcification [63,64]. Eggs are laid approximately once every 24 h in chickens [65].

#### 3.1.1. Hepatic Synthesis and Transport of Yolk Precursors

The first stage of egg formation begins in the liver, which synthesizes the major yolk precursors vitellogenin (VTG) and very low-density lipoprotein (VLDL) [66,67]. These precursors, produced under estrogen stimulation, enter the bloodstream and are selectively absorbed by developing ovarian follicles [66,67]. Genes such as *VTG1*, *VTG2*, *APOB*, *APOVLDLII*, and *VLDLR* regulate this process and influence yolk size, lipid composition, and nutrient density [68,69,70]. Receptor-mediated uptake of VLDL and VTG by the oocyte supplies essential lipids, proteins, and micronutrients required for embryonic development [71]. Genetic variation in lipid metabolism pathways (e.g., *FASN*, *SCD*, *DGAT2*) contributes to differences in yolk quality across breeds [67,72,73]. Thus, hepatic metabolic regulation forms the molecular foundation of yolk deposition and internal egg quality.

#### 3.1.2. Follicular Development and Egg Production

Follicular development plays a crucial role in regulating the reproductive performance of laying hens. However, key genes and signaling pathways involved in each stage of follicular development remain poorly understood [74]. Follicles are classified into several stages based on size, ranging from primordial follicles (diameter < 0.08 mm) to primary follicles (0.08–1 mm), undifferentiated prestratiform follicles (1–8 mm), and preovulatory follicles (>9 mm) [75]. Typically, only one dominant follicle is recruited into the preovulation hierarchy each day from a small group of follicles (6.0–8.0 mm in diameter) [74]. The growth of follicles is influenced by various factors, including the proliferation and differentiation of granulosa and theca cells. Additionally, reproductive endocrine hormones, paracrine and autocrine factors, and the hypothalamic-pituitary-gonadal axis regulate ovarian activity.

High-yielding laying hens exhibit a well-organized hierarchical follicular structure, while broiler breeders, which have lower egg production rates, exhibit poor follicular development [74]. The identification of genes affecting follicle growth, granulosa cell differentiation, and follicle senescence may provide insight into improving egg-laying performance [76].

#### 3.1.3. Calcium Ion Homeostasis and Transport in Eggshell Formation

The calcium ion transport mechanism in eggshell formation is a highly regulated physiological process, involving intestinal absorption, blood transport, and deposition in the eggshell gland. Recent research has identified several key molecular pathways and regulatory factors involved in this process [77]. Vitamin D_3_ metabolism, calcium and phosphorus homeostasis, and intestinal calcium uptake work in concert to supply the necessary components for eggshell calcification and bone mineralization.

Following ovulation, the egg enters the fallopian tube, where it is coated with proteins for approximately 3.5 h [78]. In the isthmus, the inner and outer shell membranes form. As the egg moves into the eggshell gland, calcium carbonate is deposited on the shell membrane, eventually transforming into the solid eggshell. During mineralization, calcium is mobilized from the bones of hens, accounting for 20–40% of the calcium in the eggshell. This process is crucial for the formation of a high-quality eggshell, and its efficiency decreases with the age of the hen [79]. Calcium transport involves transcellular and paracellular pathways in the intestine, where the transient receptor potential vanilloid 6 (TRPV6) channel and calcium-binding protein D28k play essential roles in calcium absorption [80].

Once calcium enters the bloodstream, it is transported in both bound and free ionic forms. Parathyroid hormone (PTH) and calcitonin (CT) maintain calcium homeostasis by regulating bone resorption and calcium reabsorption in the kidneys. In the eggshell gland, calcium ions are transported into the secretory cavity through the action of TRPV6 and Calbindin-D28k. Calcium is then pumped out of the basal membrane via the sodium-calcium exchanger (NCX1) and plasma membrane calcium ATPase (PMCA1) [80,81]. Additionally, fibroblast growth factor 23 (FGF23) has been shown to regulate calcium-phosphorus balance by affecting phosphate metabolism, which in turn influences eggshell formation [77].

### 3.2. Poultry Egg-Laying Cycle

**Figure 2:**
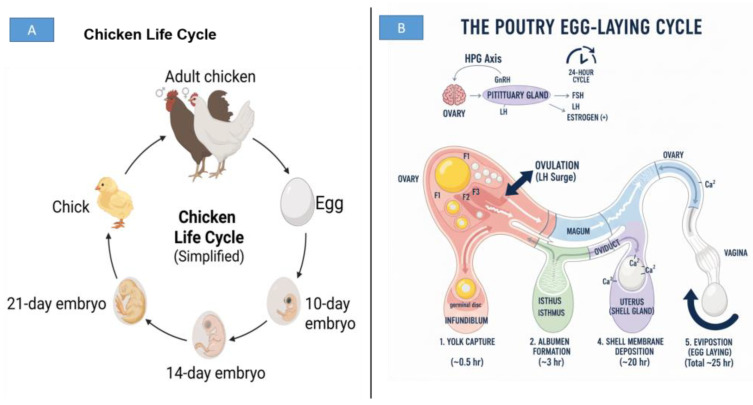
Overview of the Chicken Life Cycle and Egg-Laying Process. (**A**) Simplified representation of the chicken life cycle, illustrating the progression from egg to embryo (10-day, 14-day, and 21-day stages), chick, and adult chicken. (**B**) Schematic illustration of the poultry egg-laying cycle, showing the hormonal regulation through the hypothalamic–pituitary–gonadal (HPG) axis and the sequential stages of egg formation along the oviduct, including yolk capture in the infundibulum, albumen deposition in the magnum, shell membrane formation in the isthmus, shell calcification in the uterus, and oviposition in the vagina.

The egg-laying cycle in poultry is a coordinated physiological process governed by the hypothalamic–pituitary–gonadal (HPG) axis [82]. Light stimulation activates the hypothalamus to release gonadotropin-releasing hormone (GnRH), which subsequently induces secretion of follicle-stimulating hormone (FSH) and luteinizing hormone (LH) from the pituitary [83,84]. These hormones regulate follicular recruitment and selection within the ovary, where follicles develop hierarchically from small white follicles to large yellow pre-ovulatory follicles [85,86]. Each day, typically one dominant follicle is selected for ovulation, driven by rising estradiol and a pre-ovulatory LH surge [86,87]. Concurrently, the liver synthesizes yolk precursors such as vitellogenin and very-low-density lipoprotein (VLDL-Y), which are transported to the ovary to support yolk deposition [88]. A visual summary of the chicken life cycle and the physiological events underlying the egg-laying cycle is presented in Figure 2.

Following ovulation, the ovum enters the oviduct, where egg formation proceeds sequentially through the infundibulum, magnum, isthmus, shell gland (uterus), and vagina [89]. Fertilization—if present—occurs in the infundibulum, after which the magnum deposits albumen proteins over approximately 3 h [61,63]. The isthmus contributes the inner and outer shell membranes, while the shell gland is responsible for mineralization and deposition of the calcified shell, a process lasting 18–20 h and dependent on calcium metabolism, carbonic anhydrase activity, and uterine ion transport [90,91,92]. The completed egg is then laid through the vagina, with the entire ovulatory-ovipositional cycle taking roughly 24–26 h in commercial laying hens [61,93,94]. Variations in any step of this cycle—including hormonal profiles, nutrient supply, environmental conditions, and genetic background—directly influence egg quality traits such as shell strength, albumen height, yolk deposition, and overall structural integrity.

### 3.3. Heritability Analysis of Egg Appearance Quality

The external qualities of eggs, such as egg weight, shell thickness, shell strength, and shell color, are crucial economic traits in poultry production [95]. These traits exhibit complex quantitative characteristics that are influenced by genetic factors. Heritability, an essential parameter for understanding the genetic basis of these traits, is significantly affected by factors such as the type of trait, age, breed, and can vary dynamically over time [96]. Heritability (h^2^), which quantifies the proportion of phenotypic variation attributable to genetic factors, is commonly used in genetics studies.

#### 3.3.1. Heritability of Egg Weight and Related Traits

Egg weight has long been a primary target in breeding programs because it affects consumer preference, hatchability, chick quality, and subsequent production performance [97,98]. Genetically, egg weight is a classic polygenic trait. Studies in different chicken populations have reported a wide range of heritability estimates, generally from 0.10 to 0.75, reflecting differences in age stage, population, and analytical approach [96,98,99].

For example, a study on Mazandaran hens evaluated variance components using multi-trait, repeatability, fixed regression, and random regression models; the multi-trait model yielded h^2^ values between 0.06 and 0.41 for egg weight [99]. Another study reported age-dependent heritabilities ranging from 0.29 to 0.75, with high genetic correlations (0.72–1.00) between egg weights at different ages [100]. In Luhua chickens, heritability estimates for start-lay egg weight (start-ew) and 43-week egg weight (ew-43) were 0.30 and 0.21, respectively, confirming substantial genetic control but also age-related variation [98]. Genome-wide association studies (GWAS) across multiple time points further indicated that, except for the first egg, h^2^ for egg weight between ages generally lies between 0.47 and 0.60 [98]. For example, Ma et al. [58] observed that the heritability of egg and meat weight at 32 and 43 weeks of age was 0.42 and 0.44, respectively, illustrating how heritability can vary with age [58]. These findings suggest that egg weight is a moderately to highly heritable trait, but its expression is sensitive to age and management conditions. Genetic correlations between egg weight and other traits (e.g., body weight, egg number) can be favorable or antagonistic, which must be considered in multi-trait selection programs [101,102].

#### 3.3.2. Heritability of Eggshell Strength and Color

Eggshell strength is another key indicator that influences both the quality and commercial value of poultry eggs. Early work using acoustic resonance frequency analysis reported heritability estimates for eggshell dynamic stiffness (kdyn) ranging from 0.33 to 0.53, with a genetic correlation of 0.49 with cracking strength [103]. Subsequent analyses based on large longitudinal datasets refined these estimates: a random regression study on 2260 hens reported heritability values between 0.26 and 0.43, with genetic correlations exceeding 0.67 across test periods [104]. More recent GWAS evidence supports the polygenic architecture of shell strength; Li et al. [105] identified a heritability of 0.23 for eggshell crystal strength in an F_2_ population, indicating the importance of microstructural organization. Further research by Chen et al. [76], demonstrated that phenotypic variation in Rhode Island Red hens increased substantially after 55 weeks, while heritability declined by 22.7–81.4%, highlighting reduced genetic control in late-lay periods. Studies in Japanese quail similarly documented heritability values of 0.29–0.81, with strong genetic correlations with specific gravity, shell ratio, and shell weight [106]. Together, these findings indicate that eggshell strength has moderate-to-high heritability, but its expression is influenced by age, species, and biological stage.

Eggshell pigmentation represents another important external quality trait, driven primarily by the deposition of protoporphyrin IX, biliverdin, and their zinc chelates in the cuticle and outer calcified layers [107,108]. This pigmentation varies across breeds and individuals due to genetic, nutritional, and environmental influences [109,110,111]. Key genes such as *SLCO1B3*, *ABCG2*, and *HMOX1* regulate pigment synthesis and transport, particularly in blue and brown eggshells [112,113,114,115]. As hens age, eggshell color tends to lighten due to increased egg size and reduced pigment deposition [107,116]. Notably, darker eggshells are often associated with greater thickness and strength, enhancing hatchability and chick weight [117,118].

**Figure 3:**
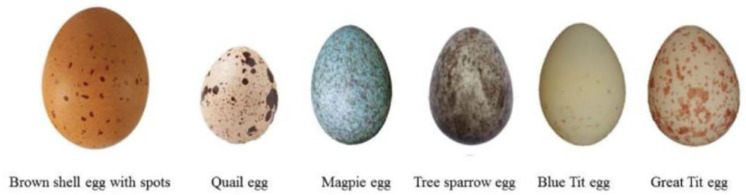
Examples of natural eggshell color variation across avian species, highlighting differences in pigmentation patterns, hue, and speckling intensity. These examples highlight the natural diversity in eggshell color and pattern among avian species, which arises from species-specific biological and environmental influences (adapted from Cheng et al., [121]).

The genetic determinism of eggshell color has been examined through numerous heritability studies. Quantitative color components such as lightness (L*), redness (a*), and yellowness (b*) show heritabilities of 0.65, 0.42, and 0.60, respectively [119]. Further evaluation of 7878 eggshell records demonstrated that luminance, redness, and yellowness exhibit similar heritability values, with luminance showing a dominant heritability estimate of 0.23, indicating meaningful contributions from dominance genetic effects [119]. These dominance effects were especially notable in blue-shelled hens. Breed-specific comparisons in Catalan poultry also revealed substantial variation, with heritability values of 0.49 in Penedesenca Negra, 0.53 in Prat Lleonada, and 0.27 in Empordanesa Roja populations [120]. Collectively, the evidence indicates that eggshell color is a medium- to high-heritability trait shaped by complex polygenic regulation across poultry breeds, as illustrated in Figure 3 through the distinct variations in color intensity, spotting patterns, and background pigmentation.

#### 3.3.3. Molecular Determinants of Eggshell Quality

The molecular regulation of eggshell quality is governed by an interconnected network of signaling pathways, ion transporters, and matrix proteins that collectively orchestrate shell mineralization. Multi-omics studies over the past five years have clarified the central role of calcium signaling in coordinating this process. During calcification, the uterus transports Ca^2+^ across the epithelial barrier and secretes matrix components that guide nucleation of calcium carbonate crystals. This mechanism depends on the continuous supply of Ca^2+^ and CO_3_^2−^ from the bloodstream, with ion movement mediated through epithelial channels and vesicle-mediated secretion [78,122]. Disruption of these finely tuned processes, such as the abnormal expression of solute carrier family members *(SLC13A1*, *SLC4A4*) and ion channel genes (*CLCN2*, *KCNJ15*), compromises the homeostasis of Ca^2+^ and HCO_3_^−^ and subsequently impairs shell mineralization [122]. Complementary GWAS analyses, such as those conducted in Sichuan White geese, have identified additional loci influencing external shell traits, including a cluster of five SNPs within an 11 bp region of the *PP4R2* gene that is significantly associated with shell thickness [119]. These findings highlight the multi-gene architecture that underpins eggshell formation and its vulnerability to perturbations in ion balance and transport.

The protein components of the eggshell matrix consist of three distinct proteomes: proteins specific to the eggshell, ubiquitous proteins present in various tissues, and eggshell-specific proteins [123,124]. Variants within these matrix protein genes directly influence shell quality; for example, the g.22,598,071 C > T missense mutation in exon 4 of *OCX-32* alters amino acid composition and modifies shell strength, specific gravity, and shell ratio. Post-translational modifications further refine matrix assembly: phosphorylated proteins such as *OC-17* facilitate crystal adhesion and growth, whereas N-glycosylated proteins like OVOT contribute to immune protection at the shell surface. Transcriptomic profiling of the uterus has identified temporal increases in genes central to mineralization, including *OC-116* and *OTOP2*, underscoring the dynamic regulation of shell deposition [106]. Importantly, recent work has demonstrated that the eggshell membrane—historically overlooked—plays a significant role in determining shell integrity. Variation in the *ABCC9* gene, particularly the rs312796152 (est_a9) intronic mutation, alters the function of ATP-sensitive potassium channels, modifies the ionic microenvironment of the shell gland, and affects tip thickness [76]. Given that the strength and thickness of eggshells are determined by the interaction of multiple layers, including the calcified and membranous components, future research should give more attention to the role of eggshell membranes in determining overall quality. To further elucidate these key genetic factors, the following table summarizes some of the significant genes and pathways involved in eggshell quality (Table 1).

#### 3.3.4. Internal Quality Heritability Analysis

The quality of egg yolk is a crucial internal factor in determining egg quality, and it is regulated by multiple genes with a measurable heritability. Studies on yolk characteristics, such as the work of Zhang et al. [126], in Hotan Black chickens, show that thermogelated yolk texture traits—including hardness, cohesion, adhesion, chewiness, and resilience—generally display low heritability (0.044–0.078), although several associated SNP loci and candidate genes have been identified. Heritability estimates for yolk quality vary widely across breeds and analytical models, indicating that genetic contributions are modulated by population structure, environmental factors, and measurement methodology. Albumen-related traits similarly demonstrate substantial genetic control. For example, Zhang et al. [126], reported moderate to high heritability for albumen height (0.51) and albumen weight (0.59) in short-sized brown-shelled layers. Further research on the entire egg-laying cycle of local egg-laying chicken breeds revealed that the heritability of protein quality-related traits ranged from 0.05 to 0.62 [127]. While the heritability values of protein quality traits vary across studies due to differences in experimental populations and analytical methods, the findings underscore the substantial genetic influence on egg protein quality. These heritability analyses provide valuable insights for the selection and breeding of poultry with enhanced egg quality traits, highlighting the importance of genetic factors in the improvement of egg yolk and egg protein quality.

## 4. The Key Genetic Factors Influencing Internal Quality

The yolk is primarily composed of lipids, which are derived from yolk precursors, including very low-density lipoprotein (VLDL) and vitellogenin (VTG), both synthesized in the liver. These lipids dissolve in the bloodstream and are subsequently transported to the ovarian follicles via the circulatory system [128]. In addition to the liver’s critical role in yolk formation, the follicle also plays a significant part in determining yolk quality during egg formation. Early in the egg formation process, a substantial amount of yolk precursors is accumulated by the follicles, which proliferate accordingly. Thus, the development and maturation of the follicles are closely linked to the synthesis and accumulation of yolk. Key genes involved in follicular growth and development also influence egg weight. For example, SYNDIG1L (also known as TMEM90A), a gene encoding a synaptic differentiation-induced 1-like protein, is involved in early follicular growth and is considered a critical genetic marker affecting egg weight [129]. Additionally, some studies suggest that endoplasmic reticulum stress induced by heat stress impairs lipid transport, subsequently affecting follicular size and, consequently, egg yolk weight [130].

Lipids and cholesterol are fundamental components of eggs (Puglisi [131]), and the regulation of lipid and cholesterol synthesis and deposition in poultry can lead to increased lipid content in egg yolks, directly impacting egg weight. The STARD8 gene, which functions in lipid transfer, plays a vital role by binding and transporting cholesterol, phospholipids, and other lipid molecules, thereby affecting intracellular lipid metabolism. The regulation of lipid balance directly influences yolk size (Clark [132]), as the gene is involved in transporting cholesterol from the cell membrane to the endoplasmic reticulum, helping maintain normal cellular cholesterol levels. Similarly, YIPF6, a gene involved in Golgi apparatus–endoplasmic reticulum vesicle transport, is crucial for yolk membrane formation.

As the primary site for synthesizing yolk precursors, the liver’s capacity to produce these precursors likely influences yolk weight. From the perspective of yolk precursor formation and transport, pathways such as fatty acid biosynthesis, folate biosynthesis, fatty acid metabolism, and triglyceride metabolism are essential for successful yolk formation [128]. For instance, a comprehensive transcriptomics and metabolomics analysis by Yan et al. [21], identified potential candidate genes and regulatory pathways related to duck egg weight, with the FANS gene showing the highest protein–protein interaction nodes. Additionally, Nikolay et al. [133], found that the FASN gene, which synthesizes fatty acids in the liver and degrades triglycerides (TG) via β-oxidation, plays a critical role in VLDL formation when TG combines with apolipoproteins, phospholipids, and cholesterol. The FASN gene and associated pathways involved in regulating adipogenesis have been highlighted as key factors influencing egg weight variability. The following table summarizes recent progress on key genes influencing the internal quality of eggs and their mechanisms of action (Table 2).

## 5. Epigenetic Regulation During Egg Formation

The external quality of eggs is influenced not only by key candidate genes and pathways but also by various epigenetic factors, including DNA modifications, gene silencing, and the roles of non-coding RNAs. These epigenetic influences are significant in explaining the variability observed in egg quality research, particularly in the context of heritability studies [134,135]. DNA methylation, one of the earliest identified epigenetic modification pathways, involves the transfer of methyl groups to the fifth carbon of cytosine, resulting in the formation of 5-methylcytosine. Despite its recognized importance, much remains to be explored regarding the epigenetic regulation of egg formation.

### 5.1. DNA Methylation and Histone Modifications in Germ Cells and Reproductive Tissues

DNA methylation is one of the best characterized epigenetic marks, involving the addition of a methyl group to the fifth carbon of cytosine to form 5-methylcytosine. Quantitative analyses of DNA methylation and histone post-translational modifications (PTMs) have begun to reveal how these marks shape egg formation in poultry [134,135]. Notably, poultry primordial germ cells (PGCs) show a distinct epigenetic profile compared with mammals. Chicken PGCs do not undergo the global DNA demethylation and reduction in histone H3 lysine 9 dimethylation (H3K9me2) typically observed in mammalian germ cells. Instead, they exhibit a loss of 5-hydroxymethylcytosine, redistribution of macroH2A, chromatin compaction, and accumulation of H3K9me3 predominantly in inactive genomic regions [136]. These features indicate early, bird-specific epigenetic programming that is likely to influence subsequent reproductive development and egg traits.

Epigenetic marks can also mediate transgenerational effects. In one study, immune stimulation of female laying hens (F0) 12 h post-fertilization reduced egg production and altered egg weight in the F1 generation [136]. This transgenerational effect was linked to DNA hypermethylation of the promoters of genes such as *CPEB3* and *RIMS2* in F1 lymphocytes, which, in turn, inhibited reproduction-related pathways. Additionally, the modification of eggshell matrix proteins plays a critical role in eggshell formation, with dephosphorylation of osteopontin, for example, inhibiting calcium carbonate precipitation [137]. This underscores the importance of both identifying the complete composition of eggshell matrix proteins and analyzing their modified structures, particularly during key stages of early development stages in poultry.

In reproductive tissues, local chromatin states regulate key genes involved in egg formation. Histone modifications, DNA methylation, and chromatin accessibility in granulosa cells and oviduct epithelium integrate endocrine signals (e.g., progesterone, estrogen) with transcriptional control of genes that affect follicular development, shell formation, and albumen synthesis. Although the number of poultry-specific studies is still limited, available data indicate that epigenetic regulation is closely intertwined with the classical hypothalamic–pituitary–gonadal axis and contributes to variation in egg production and quality.

### 5.2. Epigenetic and Post-Translational Control of Shell and Albumen Formation

Epigenetic regulation also acts at the level of the oviduct, particularly in the formation of egg white (albumen) and the eggshell matrix. In the magnum and shell gland, chromatin accessibility controls the expression of major secreted proteins, including ovalbumin (OVA), lysozyme (LYZ), and ovotransferrin (TF). Studies of histone H3 lysine 27 acetylation (H3K27ac) have shown that enhancer activity at OVA and LYZ loci directly regulates their promoter activity and transcriptional output [138]. In parallel, demethylation of a C/EBPβ enhancer element in granulosa cells can trigger a surge in progesterone, which then activates oviductal gene expression through nuclear receptor signaling [138]. These examples illustrate how histone marks and DNA methylation couple endocrine cues to the transcriptional programs that define albumen quantity and composition.

Similar principles apply to albumen proteins. Quantitative N-glycoproteomics has revealed that specific glycosylation sites, such as Asn292 in ovalbumin, are modified dynamically during embryogenesis [76]. Changes at these sites enhance antibacterial and metal-chelating properties without altering overall protein abundance, indicating that PTMs fine-tune the functional properties of albumen. These “epigenetic-like” protein modifications contribute to the protective role of egg white and may underlie variation in albumen quality traits captured by heritability analyses.

### 5.3. RNA Modifications and Epigenetic Regulation of Yolk Precursors

RNA-based mechanisms add yet another layer of regulation. RNA methylation, particularly N^6^-methyladenosine (m^6^A), has emerged as a key post-transcriptional mark affecting mRNA stability, localisation, and translation efficiency. In the oviduct, m^6^A modifications on OVA mRNA have been shown to influence translation efficiency, linking RNA methylation to albumen protein output during egg formation. Fine regulation of mRNA turnover and translation through m^6^A and other RNA marks likely contributes to temporal control of protein synthesis across the laying cycle.

Epigenetic processes in the liver and ovary similarly shape the formation of yolk. DNA methylation of genes involved in yolk precursor synthesis—such as vitellogenin (VTG) and very low-density lipoproteins (VLDL)—plays a central role in determining their expression levels. Hypomethylation of the *VLDLR* and *APOB* promoters under optimal nutritional conditions enhances lipid transport and promotes the synthesis and deposition of yolk precursors, thereby affecting yolk weight and lipid composition. Histone modifications in hepatic chromatin further integrate systemic signals (e.g., hormones, dietary factors) to regulate fatty acid biosynthesis and remodeling. Experimental evidence indicates that such epigenetic modulation can alter the fatty acid profile of egg yolks and modify their amino acid composition, with downstream effects on the nutritional quality of eggs [76]. Non-coding RNAs, including microRNAs and long non-coding RNAs, are also likely to be involved in coordinating lipid metabolism, follicular development, and oviductal secretion, although data in poultry remain relatively sparse. Integrating non-coding RNA profiles with DNA methylation, histone marks, and transcriptomic data is a key future direction for understanding how epigenetic networks shape internal egg quality.

In summary, epigenetic regulation—including DNA methylation, histone modifications, non-coding RNAs, RNA methylation, and post-translational protein modifications—modulates gene expression and protein function across germ cells, liver, ovary, and oviduct. These mechanisms integrate nutritional status, immune challenges, endocrine signals, and genetic background to coordinate oocyte development and egg quality. As multi-tissue genomics, single-cell technologies, and epigenome mapping become more widely applied in poultry, they will provide a more detailed view of these regulatory layers. Such insights will be essential for designing breeding, nutritional, and management strategies that exploit epigenetic plasticity alongside genetic variation to improve egg quality and production efficiency.

## 6. Conclusions

Recent advancements in genetic, molecular, and epigenetic research have significantly deepened our understanding of poultry egg quality, highlighting the complex regulatory networks that govern key traits. Genetic studies using tools such as genome-wide association studies (GWAS) and quantitative trait loci (QTL) mapping have identified candidate genes, including *OC-116*, *CALB1*, and *CA2* for shell formation, and *OVAL*, *SPINK5*, and *SERPINB14* for albumen quality. The heritability of egg quality traits, such as shell strength, egg weight, and yolk composition, varies across species, populations, and environmental conditions, indicating the need for more refined breeding strategies. Epigenetic mechanisms, including DNA methylation, histone modifications, and RNA methylation, add a layer of complexity by influencing gene expression and protein function, further contributing to the variability observed in egg quality traits. These insights are vital for improving breeding efficiency and the development of “functional” eggs with enhanced nutritional and commercial value.

Looking forward, precision breeding strategies that combine genetic selection, epigenetic modulation, and multi-omics technologies offer the greatest potential for improving egg quality. Key challenges remain, such as the polygenic nature of many traits, the interaction between genetic and environmental factors, and the need for a better understanding of the spatiotemporal regulation of egg formation. Future research should focus on integrating functional genomic data with epigenetic profiles to better capture the complexity of egg quality regulation. Additionally, gene-editing technologies like CRISPR/Cas9 hold promise for enhancing specific egg traits without compromising animal welfare. As single-cell genomics and epigenome mapping become more widely applied, these techniques will provide invaluable tools to refine breeding programs and optimize egg production for both quality and sustainability [125]

## Figures and Tables

**Table 1:** The key genes that affect the external quality of eggshells and their functions.

Traits	Key Genes	Function	Cite	Animal
Calcification of eggshells	*SLC13A1*, *SLC4A4 CLCN2*, *KCNJ15*	Abnormal expression leads to an imbalance in the homeostasis of Ca^2+^ and HCO_3_^−^, directly disrupting the eggshell mineralization process	[113]	Chicken
Eggshell thickness	*PP4R2*	Specific range SNPS on genes are significantly associated with eggshell thickness	[51]	Goose
The formation of eggshell matrix proteins	*0C-17*, *OC-119 OTOP2*	69phosphorylated proteins in the eggshell matrix are enriched in the cell adhesion pathway, promoting the crystallization of calcium carbonate	[125]	Chicken
Eggshell strength	*ABCC9*	rs312796152 (est_a9) in the intron region regulates the ionic environment of eggshell glands by influencing the function of ATP-sensitive potassium channels, thereby leading to differences in tip thickness.	[76]	Chicken

**Table 2:** Key genes influencing internal egg quality (yolk and albumen) and their functions.

	Key Genes	Function	Cite	Animals
Egg yolk formation	SYNDIG1L	It participates in the early growth of follicles and affects the weight of the egg	[126,127,129]	Duck
Egg white protein	FASN	It synthesizes fatty acids in the liver, degrades triglycerides through β-oxidation, participates in the formation of VLDL, and affects the difference in egg weight	[133]	Duck

## Data Availability

No new data were created or analyzed in this study.
